# Neuronal organization of olfactory bulb circuits

**DOI:** 10.3389/fncir.2014.00098

**Published:** 2014-09-03

**Authors:** Shin Nagayama, Ryota Homma, Fumiaki Imamura

**Affiliations:** ^1^Department of Neurobiology and Anatomy, The University of Texas Medical School at HoustonHouston, TX, USA; ^2^Department of Pharmacology, Pennsylvania State University College of MedicineHershey, PA, USA

**Keywords:** structure of olfactory bulb, cell type, layer formation

## Abstract

Olfactory sensory neurons extend their axons solely to the olfactory bulb, which is dedicated to odor information processing. The olfactory bulb is divided into multiple layers, with different types of neurons found in each of the layers. Therefore, neurons in the olfactory bulb have conventionally been categorized based on the layers in which their cell bodies are found; namely, juxtaglomerular cells in the glomerular layer, tufted cells in the external plexiform layer, mitral cells in the mitral cell layer, and granule cells in the granule cell layer. More recently, numerous studies have revealed the heterogeneous nature of each of these cell types, allowing them to be further divided into subclasses based on differences in morphological, molecular, and electrophysiological properties. In addition, technical developments and advances have resulted in an increasing number of studies regarding cell types other than the conventionally categorized ones described above, including short-axon cells and adult-generated interneurons. Thus, the expanding diversity of cells in the olfactory bulb is now being acknowledged. However, our current understanding of olfactory bulb neuronal circuits is mostly based on the conventional and simplest classification of cell types. Few studies have taken neuronal diversity into account for understanding the function of the neuronal circuits in this region of the brain. This oversight may contribute to the roadblocks in developing more precise and accurate models of olfactory neuronal networks. The purpose of this review is therefore to discuss the expanse of existing work on neuronal diversity in the olfactory bulb up to this point, so as to provide an overall picture of the olfactory bulb circuit.

## INTRODUCTION

Our environment is filled with odorant molecules, and our emotions, moods, and even behaviors can be controlled by olfactory stimuli. We are predisposed to discriminate between more than 10^12^ odors (Bushdid et al., 2014), in part due to the variety of odorant receptors that bind to different odorants with unique affinity profiles (Malnic et al., 1999). These odorant receptors are expressed by olfactory sensory neurons in the olfactory epithelium. Since the first rat odorant receptor was cloned in 1991, approximately 400 and 1000 different functional odorant receptors have been identified in the human and mouse genome, respectively (Buck and Axel, 1991; Zhang and Firestein, 2002; Nei et al., 2008; Adipietro et al., 2012). Because each olfactory sensory neuron expresses only a single odorant receptor, different odorants can activate distinct subsets of olfactory sensory neurons. Information from activated neurons is first transmitted to the olfactory bulb. Several reviews have summarized the elaborate neuronal network that extends from the olfactory epithelium to the olfactory bulb (Wilson and Mainen, 2006; Zou et al., 2009; Sakano, 2010; Mori and Sakano, 2011; Murthy, 2011; Lodovichi and Belluscio, 2012). In the olfactory bulb, multiple types of neurons form sophisticated networks to process information before transmitting it further to the olfactory cortex. Currently, there is a pressing demand for understanding the numerous neuronal types and networks to elucidate the mechanism(s) of olfactory information processing in the olfactory bulb.

**FIGURE 1:**
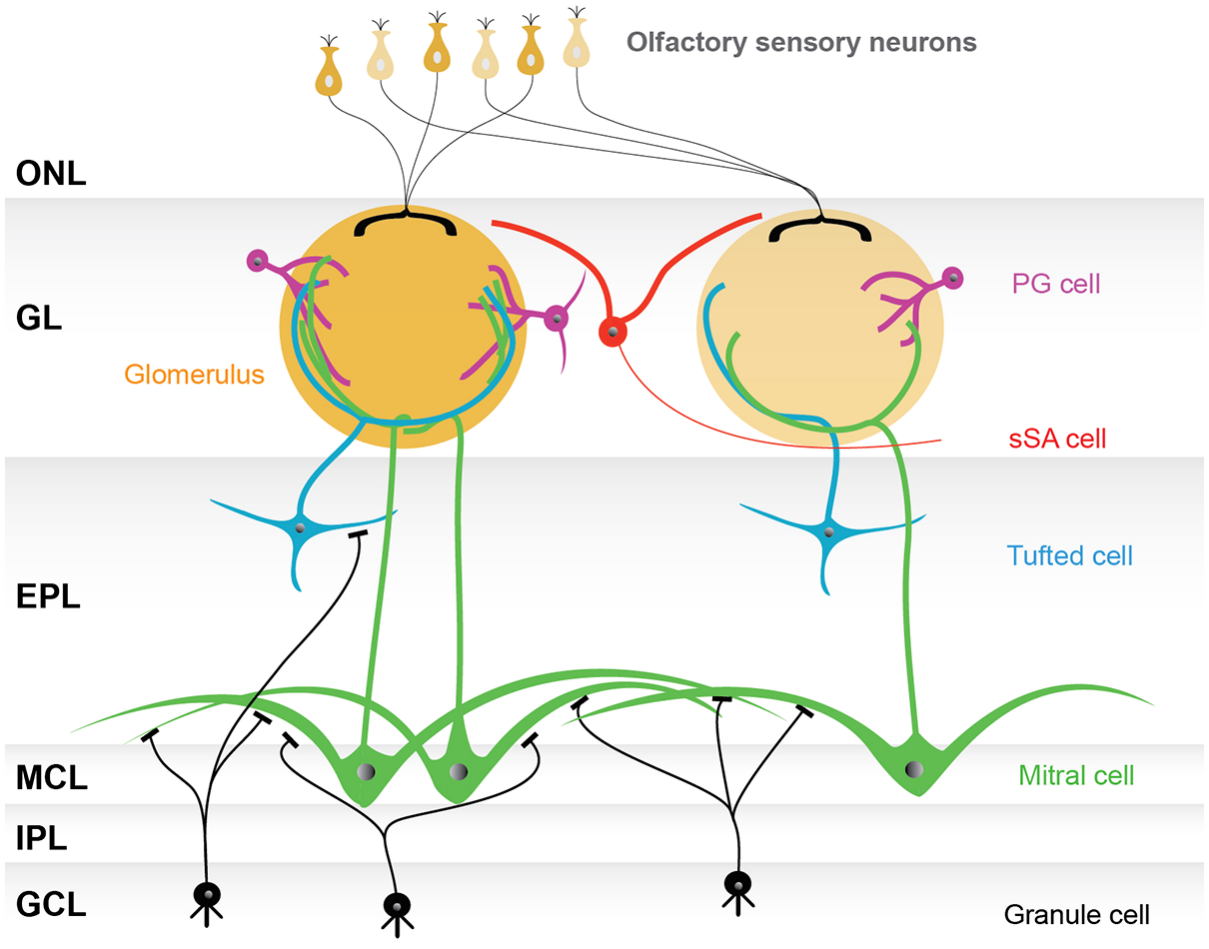
**Basic model of the olfactory bulb network.** The illustrated olfactory bulb network is based on the conventional categorization of participating neurons. The axons of olfactory sensory neurons make synapses in the glomerular layer (GL), consisting of spherical structures called glomeruli. Although there are several thousand glomeruli at the surface of the rodent olfactory bulb, olfactory sensory neurons expressing the same type of odorant receptor converge their axons into only a few glomeruli, and thus each glomerulus represents a single odorant receptor. Neurons surrounding glomeruli in the GL are called juxtaglomerular cells (JG cells), consisting of three morphologically distinct cell types: periglomerular (PG) cells, external tufted (ET) cells (not shown), and superficial short-axon (sSA) cells. There are two types of projection neurons, the mitral cells and the tufted cells, which send their axons to the olfactory cortex. The somata of mitral cells are located in the mitral cell layer (MCL), while the tufted cells are scattered throughout the EPL. Both mitral and tufted cells project a single primary dendrite into a single glomerulus, where they receive synaptic inputs from the axons of olfactory sensory neurons and make reciprocal synapses with the dendrites of PG cells. Secondary dendrites of mitral and tufted cells are elongated in the external plexiform layer (EPL), where reciprocal synapses are formed with granule cell dendrites. The internal plexiform layer (IPL), in which axons from mitral cells and axon collaterals of ET cells run, and the granule cell layer (GCL), which is largely composed of granule cells, both lie beneath the MCL. Granule cells are axon-less interneurons extending dendrites apically into the EPL. Abbreviation: ONL, olfactory nerve layer.

Histologically, the olfactory bulb is divided into multiple layers. Intensive Golgi analyses in the 1970s succeeded in visualizing the morphology of neurons in each layer, and showed that the distinct layers were composed of morphologically distinct cells (Price and Powell, 1970a,b; Pinching and Powell, 1971a). Therefore, the neurons in the olfactory bulb have conventionally been categorized based on the layers in which their cell bodies are found. According to this categorization, juxtaglomerular (JG) cells, mitral cells, tufted cells, and granule cells were first defined. JG cells are now known to include three morphologically distinct cell types, the periglomerular (PG) cells, external tufted (ET) cells, and superficial short-axon (sSA) cells. This categorization provides us with a basic model of the olfactory bulb network (**Figure 1**).

However, we are far away from forming a comprehensive model of the olfactory bulb network. An increasing number of studies suggest that conventionally categorized neurons in the brain comprise heterogeneous populations, and that the neuronal types in the olfactory bulb are among the most diverse (Shipley and Ennis, 1996). For example, PG cells are molecularly heterogeneous (Kosaka et al., 1995; Kosaka and Kosaka, 2005, 2007a; Panzanelli et al., 2007; Parrish-Aungst et al., 2007), and subgroups of mitral cells with different morphological and/or electrophysiological properties have been identified (Padmanabhan and Urban, 2010; Angelo et al., 2012). Furthermore, granule cells can be separated into morphologically distinct subgroups (Mori et al., 1983; Orona et al., 1983), and divergent properties between granule cells generated during developmental and adult stages are noted (Lemasson et al., 2005). In addition, some olfactory bulb neurons have not been typically included in conventional categories due to their relatively small numbers [e.g., short-axon cells in the External plexiform layer (EPL) and the granule cell layer (GCL)]. Nonetheless, recent technological advances now make it possible to target and analyze even these miniscule cell populations. Moreover, new neuronal types and connections in the olfactory bulb continue to be discovered (Merkle et al., 2014).

Nevertheless, new neuronal types and connections are often not taken into account in building a model of the olfactory bulb network. We are concerned with this trend of omission as it may hinder future progress in research. The major problem is that novel findings are scattered across many literary references, and recent reviews have not effectively summarized the neuronal diversity in the olfactory bulb. Here, we gather the scattered results and summarize the discoveries regarding the new neuronal types and connections in each layer. Since understanding the inputs and outputs of neurons is fundamental to building a network model, we focus mainly on somata locations, axon/dendrite extension patterns, neurotransmitters, and/or the physiological properties of neurons. We believe that this review greatly adds to our knowledge of the general model of the olfactory bulb network, and brings the information about this brain region to another level. In this article, we focus on neurons rather than on glia and the rodent main olfactory bulb rather than accessory olfactory bulb. The authors apologize to those whose work was not included here due to space limitations.

## GLOMERULAR LAYER

### NEURONS IN THE GLOMERULAR LAYER AND THEIR MORPHOLOGY

Neurons in the glomerular layer (GL) are morphologically heterogeneous and are of three identified types, PG cells, sSA cells, and ET cells (Pinching and Powell, 1971a). Generically, they may also be referred to as JG cells when their morphological type is not specified. Most of these cells (PG cells, sSA cells, and a portion of the ET cells) are actually interneurons and do not innervate brain regions outside the olfactory bulb.

The PG cell is the most abundant type of neuron in the GL (Parrish-Aungst et al., 2007). These cells have the smallest cell body (5–10 μm in diameter) among the three morphological types. PG cells typically project their dendrites to a single glomerulus, and only occasionally to multiple glomeruli. Their dendrites ramify in a smaller portion of the glomerulus than the dendrites of ET cells. PG cells are generally thought to bear an axon (Pinching and Powell, 1971a), but axonless subtypes may also exist (Kosaka and Kosaka, 2011). The length of the axon is variable, and can extend as far as 5–6 glomeruli (∼600 μm; Pinching and Powell, 1971a). Axons of PG cells terminate in the interglomerular space.

The sSA cell was first reported by Pinching and Powell (Pinching and Powell, 1971a). The percentage of sSA cells among JG cells is thought to be small, although no estimate has been provided. The somata of sSA cells are slightly larger than those of PG cells, at 8–12 μm. These cells have dendrites that course exclusively in the interglomerular space, with an axon that extends as far as 1–2 glomeruli (Pinching and Powell, 1971a,c). No dendrodendritic connections have been found among this population (Pinching and Powell, 1971b,c). However, some recently described sSA cells have a somewhat distinct morphology from previously described sSA cells (Aungst et al., 2003; Kiyokage et al., 2010). This recently described population will be discussed in detail below.

The ET cell has the largest cell soma among the three types of JG cells, at 10–15 μm (Pinching and Powell, 1971a). The primary dendrites of ET cells are generally mono-glomerular, with a small subpopulation being di-glomerular (Ennis and Hayar, 2008). In contrast to PG cells, ET cell dendrites occupy a large volume of the glomerulus. To date, at least two morphologically distinctive subgroups of ET cells have been reported (Macrides and Schneider, 1982; Schoenfeld et al., 1985). One group has no secondary dendrites; the cell body is only found in the GL; and the axon is apparently restricted within the olfactory bulb. The other group of ET cells has secondary dendrites that extend to the EPL, with cell bodies generally found in the deeper one-third of the GL, or in the EPL near the boundary with the GL. The axons of the latter group project either to the internal plexiform layer (IPL) of the other side (medial-lateral) of the same bulb, or to the anterior olfactory nucleus (AON) pars externa (pE). The AONpE connects the circuits associated with homotypic glomeruli receiving input from olfactory sensory neurons that express the same odorant receptor between right and left olfactory bulb (Liu and Shipley, 1994; Lodovichi et al., 2003; Yan et al., 2008). As discussed later, these two groups may also be distinct in other attributes.

**FIGURE 2:**
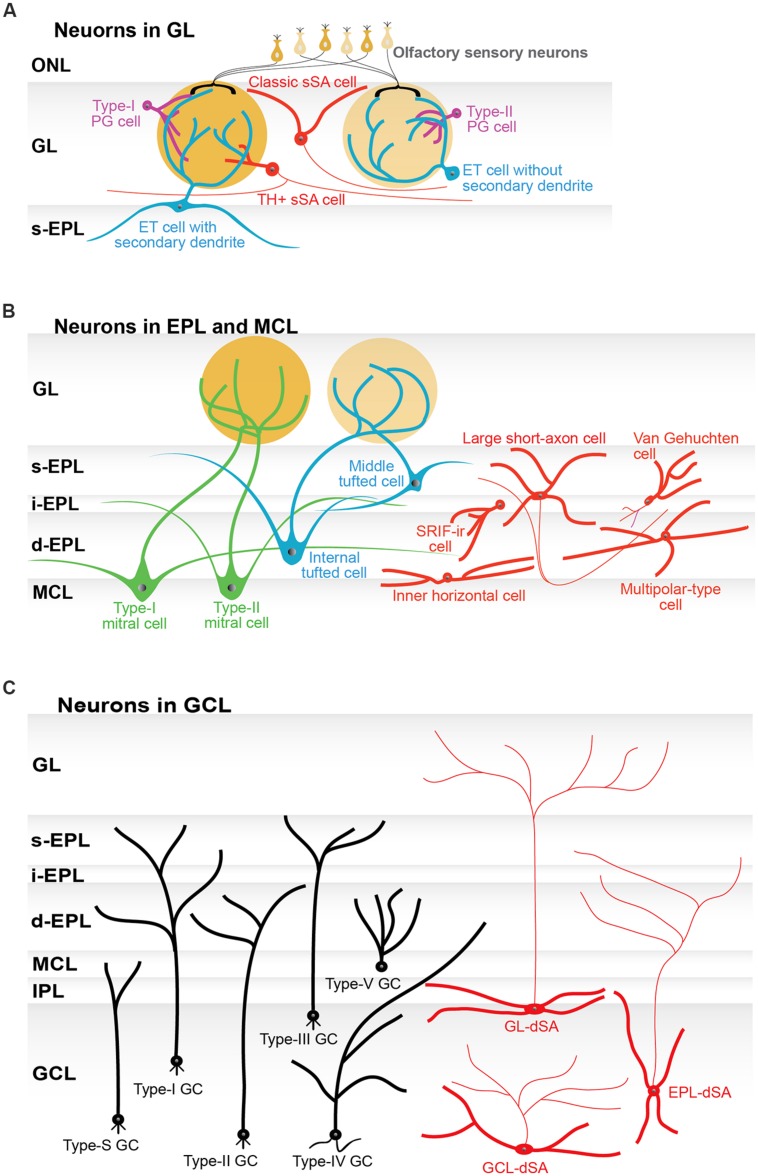
**Subtypes of neurons in the olfactory bulb. (A)** Schematic illustration of the subtypes of periglomerular cells (PG cells; purple), superficial short-axon cells (sSA cells; red), and external tufted cells (ET cells; blue). Two subtypes of PG cells are based on their synaptic connections. Type-I PG cells receive synaptic inputs on their dendrites from both olfactory sensory neurons and neurons in the olfactory bulb. Type-II PG cells only receive inputs from neurons in the olfactory bulb. PG cells can be further divided into neurochemical subtypes (not shown in the figure). Classically, sSA cells had an axon and dendrites. The dendrites did not enter a glomerulus. More recently reported types of sSA cells are positive for tyrosine hydroxylase (TH) and connected to a few to tens of glomeruli. Subtypes of ET cells are determined by morphology: those without and those with secondary dendrites. The ET cells with secondary dendrites are more frequently found around the border between the GL and the superficial external plexiform layer (s-EPL). **(B)** Schematic illustration of subtypes of tufted cells (green) and mitral cells (blue), as well as interneurons (red) in the external plexiform layer (EPL) and the mitral cell layer (MCL). Tufted cells are classified as external, middle, and internal tufted cells based on their soma location. ET cells are discussed in the GL section of the main manuscript and thus are not shown here. The EPL itself can be divided into multiple sub-layers, including the s-EPL, the intermediate EPL (i-EPL), and the deep EPL (d-EPL). The two subtypes of mitral cells are based on the depth of their secondary (basal) dendrites in the EPL sub-layers and are referred to as type-I and type-II mitral cells, respectively. On average, middle tufted cells have smaller cell somata than internal tufted cells, and internal tufted cells have smaller cell somata than mitral cells. Among interneurons, only large short-axon cells have axons. Some of the neurons that had originally been categorized as short-axon cells have an axon-like process (see main text), but they are not shown in this figure to avoid any confusion. Van Gehuchten cells are axonless cells, while inner horizontal and multipolar-type cells are identified by their morphological features and locations. The majority of interneurons have been studied by parvalbumin (PV) labeling. In addition, somatostatin-immunoreactive (SRIF-ir) cells are located in the i-EPL/d-EPL and extend their dendrites specifically to the d-EPL. **(C)** Schematic illustration of six subtypes of granule cells (GC; black) and three subtypes of deep short-axon cells (dSA cells; red) in the granule cell layer (GCL). The subtypes of granule cells and dSA cells are determined based on the location (depth) of their cell somata in the GCL and the layer or sublayer to which their dendrites extend. These subtypes of GC are referred to as type-I, type-II, type-III, type-IV, type-V, and type-S cells. The subtypes of dSA cells are referred to as GL-dSA, EPL-dSA, and GCL-dSA cells, reflecting the distribution of their axons. Abbreviations: GL, glomerular layer, ONL, olfactory nerve layer; IPL, internal plexiform layer.

These JG neurons, including the subtypes discussed in the following subsections, are summarized in **Figure 2A** and **Table 1**. More detailed description about basic neuronal circuits in the GL can be found in an excellent review by Wachowiak and Shipley (2006).

**Table 1:** Glomerular layer.

Cell type	Periglomerular cell (PG cell)		Superficial short-axon cell (sSA cell)		External tufted cell (ET cell)	

Subtype	Type-I	Type-II	Classic	TH+/GAD67+	No secondary dendrite	With secondary dendrite
Soma size	5–10 μm		8–12 μm		10–15 μm	

Soma location	Interglomerular space		Interglomerular space		Interglomerular space	
						GL-EPL border

Dendrite extension	Single glomerulus (infrequently two glomeruli)		Interglomerular space	Multiple glomeruli	Single glomerulus (infrequently two glomeruli)	
						Superficial EPL

Input	ET cell, PG cell, sSA cell, tufted cell, mitral cell, GL-dSA cell, centrifugal fiber		PG cell, sSA cell, centrifugal fiber	ET cell (same glomerulus)	OSN, ET cell (VGLUT3+, same glomerulus)	
	OSN					GC

Output	Tufted cell, mitral cell, PG cell, OSN		PG cell, sSA cell (classic), tufted cell, mitral cell	ET cell (other glomeruli)	PG cell, sSA cell (TH+/GAD67+), ET cell (same glomerulus), mitral cell, tufted cell	
						neuron in AONpE

Transmitter	GABA, dopamine (in the TH+ subtype)		*Unknown*	GABA, dopamine	Glutamate, GABA (in the VGLUT3+ subtype)	
						CCK, vasopressin

Known neurochemical subtypes	TH+	CB+, CR+	*Unknown*	*Unknown*	VGLUT2+, VGLUT3+	

Other known molecules expressed (in subpopulation)	GAD65, GAD67, PV, neurocalcin, GABA_A_-R α5 subunit		*Unknown*	GAD67, TH	CB, GAD67, GABA_A_-R α1 and α3 subunits	
						CCK, vasopressin

Proportion to the total JG cell population	Majority		*Unknown* (minority)	Less than 10%*	*Unknown*	

Function	Inhibition within the glomerulus		*Unknown*	Inhibition across glomeruli	Excitation within the glomerulus	
						Connecting circuits associated with the glomeruli of the same ORN

Additional notes				May have been included in TH-positive PG cell in some studies		Also referred to as “superficial tufted cell”

References	Pinching and Powell (1971a), Pinching and Powell (1971c), Kosaka et al. (1998), Kosaka and Kosaka (2005), Wachowiak and Shipley (2006), Kosaka and Kosaka (2007a), Parrish-Aungst et al. (2007), Panzanelli et al. (2007), Shao et al. (2009)		Pinching and Powell (1971a), Pinching and Powell (1971c)	Aungst et al. (2003), Kiyokage et al. (2010)	Macrides and Schneider (1982), Panzanelli et al. (2005), Tatti et al. (2014)	
						Liu and Shipley (1994), Tobin et al. (2010)

*TH+ neurons (TH+ PG cell and TH+ sSA cell) represent ~10% of entire JG cell population (Parrish-Aungst et al., 2007).

### DIVERSITY OF PERIGLOMERULAR CELLS REGARDING SYNAPTIC ORGANIZATION

PG cells can be divided into two subtypes based on its synaptic organization. A glomerulus in the olfactory bulb is composed of two distinct kinds of anatomical compartments (Kosaka et al., 1996, Kasowski et al., 1999). One compartment includes the processes of olfactory sensory neurons (ON zone), and the other compartment lacks these processes and is instead occupied by the processes of bulbar neurons (non-ON zone). Some PG cells extend their dendrites to both zones (referred to as type-I PG cells), but other PG cells extend their dendrites only to the non-ON zone (referred to as type-II PG cells; Kosaka et al., 1998). This observation suggests that type-II PG cells do not receive direct inputs from olfactory sensory neurons.

In parallel with the above-described anatomical heterogeneity, experiments using slice preparations suggested two types of synaptic organization in PG cells, based on their physiological properties (Shao et al., 2009; Kiyokage et al., 2010). The first type of neuron receives single spontaneous excitatory post-synaptic currents (EPSCs) and exhibits consistent and shorter delays in response to the electric stimulation of olfactory nerve bundles in the ONL. The second type of neuron receives a burst of spontaneous EPSCs and exhibits longer and varying delays to peri-threshold nerve stimulation. The former neuron is likely driven by direct monosynaptic inputs from olfactory sensory neurons (ON-driven cells), whereas the latter is likely driven by polysynaptic inputs through olfactory bulb neurons, such as ET cells (ET-driven cells; Shao et al., 2009).

It is tempting to relate type-I and type-II PG cells to ON-driven cells and ET-driven cells, respectively. However, the relationship may not be that straightforward. Type-II PG cells could be exclusively ET-driven, given that they have no connection to olfactory sensory neurons. On the other hand, type-I PG cells, which may connect to both olfactory sensory neurons and other cells, could in principle be driven by either pathway. Therefore, the dominant pathway is probably determined by as yet unidentified, additional factors (Kiyokage et al., 2010).

### DIVERSITY OF PERIGLOMERULAR CELLS REGARDING NEURONAL TRANSMITTER

The neurotransmitters of PG cells are described largely on the basis of expression of related molecular markers. Among the many candidate markers related to neurotransmitters (Kosaka et al., 1998), glutamic acid decarboxylase (GAD), and tyrosine hydroxylase (TH) have attracted the most attention. Two isoforms of GAD (GAD65 and GAD67) are of particular interest. First, the expression of these proteins in a neuron implies its physiological function as a GABAergic neuron. Second, one or both of these isoforms is expressed in more than half of the entire JG cell population (Parrish-Aungst et al., 2007; Whitman and Greer, 2007). Even though the relative proportions of each isoform in a particular cell and/or their co-expression are of broad interest, no conclusive figures are yet available. One technical difficulty may be a potential mismatch between the population positive for GAD immunolabeling, genetic markers [e.g., green fluorescent protein (GFP)], and the true population of GABAergic neurons. Another problem is that a fraction of the PG cell population is not labeled by the general neuronal marker, NeuN (Panzanelli et al., 2007; Parrish-Aungst et al., 2007; Whitman and Greer, 2007). These limitations make it difficult to reliably estimate the number of all PG cells and their expression of GAD.

TH is an essential enzyme for the synthesis of dopamine. TH-positive cells account for approximately 10% of all JG cells, and most of these, if not all, co-express GAD. Indeed, the majority of the cells, and perhaps the entire population, is positive for GAD67, while a minor percentage is positive for both GAD65 and GAD67, or possibly for GAD65 alone. TH-positive neurons were considered as belonging to the PG cell population (Kosaka et al., 1998; Kosaka and Kosaka, 2005). However, recent studies revealed that some of TH-positive neurons are in fact morphologically similar to sSA cells (see below).

### MOLECULAR DIVERSITY OF PERIGLOMERULAR CELLS

A number of studies address the molecular diversity of JG cells (Panzanelli et al., 2007; Parrish-Aungst et al., 2007). Although many of these studies did not originally intend to focus on specific morphological cell types, ET cells are negative for most of the molecular markers employed in these studies (but see below). Other studies limited their analyses to only the GABAergic neurons. As a consequence, while the vast majority of these studies intended to address the molecular diversity of PG cells, they might have inadvertently included GABAergic sSA cells and/or GABAergic ET cells.

The calcium-binding proteins, and particularly calretinin (CR) and calbindin (CB), have been extensively studied for their expression in PG cells. The expression of CR, CB, and TH is mutually exclusive, suggesting that each of these markers corresponds to a specific subtype of PG cell. Nearly all TH-positive and CB-positive PG cells, as well as the majority of CR-positive PG cells, are positive for GAD (Kosaka and Kosaka, 2007a; Sawada et al., 2011). TH, CB, and CR are co-expressed in PG cells with both GAD isoforms, and therefore are not exclusively associated with any one isoform. TH-positive PG cells are type-I PG cells, while CB-positive and CR-positive PG cells are type-II PG cells (Kosaka and Kosaka, 2005, 2007a). Multiple research groups have made attempts to determine the proportions of subtypes expressing these neurochemicals in the mouse (Panzanelli et al., 2007; Parrish-Aungst et al., 2007; Whitman and Greer, 2007; Sawada et al., 2011). Although the results are not ideal for direct comparisons between studies, the percentages of each marker fall within certain ranges: 30.0–44.0% for CR, 12.6–20.0% for TH, and 9.8–15.0% for CB. The difficulty of direct comparison mainly stems from varying definitions of overall cell populations. As described previously, enumeration of PG cells is technically challenging. Each laboratory has taken different paths to estimate the overall population of PG cells in the olfactory bulb, including counting all neurons positive for any neuronal markers tested (Parrish-Aungst et al., 2007); counting all cells labeled with the fluorescent dye DRAQ-5, except for ET cells (Whitman and Greer, 2007); counting cells immunohistochemically positive for a mixture of anti-GAD65/67 and anti-GABA (Kosaka and Kosaka, 2007a); or using transgenic mice, such as GAD67-GFP knock-in mice (Panzanelli et al., 2007) or vesicular GABA transporter (VGAT)-venus mice (Sawada et al., 2011), in which GABAergic neurons are labeled with fluorescent proteins.

The expression of additional neurochemical markers, such as neurocalcin, parvalbumin (PV), and GABA_A_ receptor α5 subunit, have also been explored in several studies (Panzanelli et al., 2007; Parrish-Aungst et al., 2007; Whitman and Greer, 2007). Although these markers have not been investigated as extensively as the molecular markers, CR, CB, and TH, no evidence for co-expression with CR, CB, TH, or any other co-tested markers has been found. Accordingly, PG cells positive for some of these markers may also represent distinct cell subtypes. There are also reports of other markers that may only partially co-localize in PG cells (Kosaka and Kosaka, 2012). Such studies imply that the number of cellular molecular subtypes will continue to grow in the future based on the combination of multiple molecular markers. However, expression of many molecular markers (e.g., calcium-binding proteins) does not suggest the function of the cell subtype in and of themselves. Functional characterization of each cell subtype will therefore be essential.

### DIVERSITY OF SUPERFICIAL SHORT-AXON CELLS

There is little literature explicitly describing the diversity of sSA cells. However, two types of sSA cells have apparently been documented in the literature. The first type is the sSA cells described by Pinching and Powell (Pinching and Powell, 1971a; see above). Here we mainly discuss the second type that was reported more recently.

Retrograde tracing showed that a subpopulation of JG cells possess long neuronal processes (several 100 μm to 1 mm). This population of neurons is positive for both TH and GAD67 (TH+/GAD67+ neurons; Kiyokage et al., 2010; Kosaka and Kosaka, 2011). Kiyokage et al. (2010) proposed that interglomerular process-bearing TH+/GAD67+ neurons might be classified as sSA cells. These cells were once reported as glutamatergic sSA cells by the same group (Aungst et al., 2003).

However, the morphology of this proposed TH+/GAD67+ sSA cell is clearly distinct from that of the “classic” sSA cell reported by Pinching and Powell (Kosaka and Kosaka, 2011; Pinching and Powell, 1971a,c). A TH+/GAD67+sSA cell has an axon that extends for ∼1 mm, and its dendrites make contacts with up to 50 glomeruli. On the other hand, a classic sSA cell has an axon that extends for just one to two glomeruli, and its dendrites avoid glomeruli. This discrepancy suggests that TH+/GAD67+sSA cells are a different breed from the classic sSA cell, even though their morphology is typical of the short-axon cell. It seems possible that there are two (or more) types of short-axon cell-like neurons in the GL, considering the heterogeneity of short-axon cells in the EPL and the GCL, as discussed in more detail later in the review.

Finally, TH-positive JG cells show further heterogeneity in soma size, where the size forms a bimodal distribution (Pignatelli et al., 2005; Kosaka and Kosaka, 2008b). The “small-soma” group is typical of PG cells, whereas the “large-soma” group exhibits a slightly larger soma than the PG cells. It is worthwhile to point out that TH+/GAD67+sSA cells are included in the large-soma group (Kosaka and Kosaka, 2008b). Therefore, we suggest that TH+/GAD67+ cells account for only a subpopulation of TH-positive JG cells.

### MORPHOLOGICAL DIVERSITY OF EXTERNAL TUFTED CELLS

As discussed previously, ET cells are historically divided into two morphologically distinct subtypes: those without and those with secondary (basal) dendrites (alternatively, the cells can be divided into three subtypes by further dividing the dendrite-bearing cells according to morphological differences in the secondary dendrites; Macrides and Schneider, 1982). More recently, these morphological differences were shown to correlate with physiological distinctions (Antal et al., 2006). Therefore, the non-basal-dendrite-bearing and basal-dendrite-bearing ET cells may form two (or potentially more) separate populations.

In several studies, the basal-dendrite-bearing cells are referred to as superficial tufted cells (Ezeh et al., 1993; Kiyokage et al., 2010). Note that tufted cells are most typically classified into three types: external, middle, and internal tufted cells (see below). On the other hand, even though some ET cells with secondary dendrites are located in the superficial part of the EPL, they can still be distinguished from the middle tufted cells, particularly because few axons from these cells travel beyond the AON. It is therefore of great interest to determine whether the synaptic organization within the glomerulus is the same or different among each subtype of ET cells and the middle tufted cells.

### DIVERSITY OF EXTERNAL TUFTED CELLS REGARDING NEURONAL TRANSMITTER

ET cells have long been considered exclusively glutamatergic. However, the latest study has revealed a novel and interesting subtype of ET cells, which is identified by its expression of vesicular glutamate transporter (VGLUT)3. A portion of this subtype is found to be not only glutamatergic but also GABAergic (Tatti et al., 2014). The synaptic targets of ET cells include other JG cells and projection neurons (both tufted and mitral cells) in the same glomerulus that connect through dendrodendritic synapses. For projection neurons, ET cell mediated di-synaptic inputs are a major source of excitatory inputs, in addition to direct inputs from olfactory sensory neurons (Wachowiak and Shipley, 2006; Najac et al., 2011; Gire et al., 2012). VGLUT3-positive subtype has been exhibited unique connectivity, that is, it excites tufted cells but not mitral cells. VGLUT3-positive subtype also inhibits the other subtype of ET cells in the same glomerulus (Tatti et al., 2014).

Furthermore, to date, two peptide hormones are known to be released from subpopulations of ET cells. Liu and Shipley (1994) reported a subpopulation of ET cells (referred to as “superficially situated tufted cells”) that are involved in intrabulbar (medial-lateral) connections by using the peptide hormone, cholecystokinin (CCK), as a neurotransmitter. However, it is not clear whether CCK is used only by this type of ET cell, or by other ET cells as well. Tobin et al. (2010) reported a subpopulation of ET cells that releases a peptide hormone vasopressin. These vasopressinergic ET cells are involved in processing olfactory signal related to social recognition. These CCKergic and vasopressinergic ET cells are most often located around the boundary of GL and EPL, and have secondary dendrites.

### MOLECULAR DIVERSITY OF EXTERNAL TUFTED CELLS

The molecular diversity of ET cells began to be revealed recently. For example, ET cells can be divided into specific subpopulations based on immunoreactivity for different GABA_A_ receptor subunits, such as the α1 and α3 subunits (Panzanelli et al., 2005). Another study has classified ET cells into VGLUT2-positive and VGLUT3-positive subpopulations. VGLUT2-positive and VGLUT3-positive subpopulations are also distinctive about the synaptic organization in the glomerulus (Tatti et al., 2014). In the latter study, a portion of VGLUT3-positive ET cells coexpressed CB, but few of them coexpressed CR or TH. Although these molecules may not be strictly specific as molecular markers, they would be enormously helpful for the future studies on ET cells.

### ADULT-BORN NEURONS IN THE GLOMERULAR LAYER

Subpopulations of olfactory bulb neurons are generated during adulthood and continue to be replaced throughout the life of the organism. JG cells are included in these subpopulations, although the proportion of adult-born neurons in the GL is much lower than that in the GCL. These neurons are generated in subventicular zone (SVZ) or rostral migratory stream, and then migrate to the olfactory bulb through the rostral migratory stream. Adult-born JG cells are probably primarily composed of PG cells, based on their morphology. Whitman and Greer (2007) described two morphologically distinct adult-born JG cells, one with a dendritic arbor limited to one or two glomeruli, and the other with more extensive multi-glomerular dendrites. The latter cells also had slightly larger cell bodies. Nonetheless, TH-positive neurons with interglomerular processes (i.e., the TH+/GAD67+ sSA cells discussed earlier) may not be generated in adulthood (Kosaka and Kosaka, 2009).

As in the overall PG cell population, neurochemical subtypes have been revealed in adult-born JG cells. The vast majority of these subtypes are likely GABAergic (Whitman and Greer, 2007; Sawada et al., 2011). However, a recent study reported the presence of adult-born, glutamatergic JG cells (Brill et al., 2009). Even though their numbers are small, further characterization of these neurons would be of great interest.

Regarding other molecular markers, adult-born PG cells express all three typical molecular markers discussed above: TH, CR, and CB. Whitman and Greer (2007) determined the percentage of each PG cell subtype in adult-born JG cells of a specific age by using the thymidine analog, 5-bromo-2′-deoxyuridine (BrdU), to label cells generated at the time of injection. At 46 days post-BrdU injection, a date at which adult-born PG cells are morphologically mature, BrdU-positive cells (∼46 days old) contain higher percentages of TH-positive, CR-positive, and GAD67-positive neurons compared with BrdU-negative cells (cells of all other ages, including embryonic cells). This finding raises the possibility that the relative percentage of PG cell subtypes may not be static, but instead keeps changing throughout life.

## EXTERNAL PLEXIFORM LAYER AND MITRAL CELL LAYER

### PROJECTION NEURONS IN THE EXTERNAL PLEXIFORM LAYER AND MITRAL CELL LAYER

Odor signals, which are processed within the glomerulus, propagate in the EPL along the primary dendrite of two types of projection neurons, mitral and tufted cells (**Figure 1**). The signal arrives at the cell body of tufted cells in the EPL and mitral cells in the mitral cell layer (MCL), then horizontally back-propagates through the secondary dendrites in the EPL. The back-propagation signal is considered not to be attenuated, but rather to be conducted throughout the dendrites until it is blocked by local inhibition from granule cells (Xiong and Chen, 2002). The horizontal signal propagation is believed to inhibit the activity of other mitral/tufted cells via the dendrodendritic interaction with granule cells and other interneurons. This process is called lateral inhibition. The EPL is mostly occupied by dendritic fibers that are secondary dendrites of mitral/tufted cells and apical dendrites of granule cells.

Mitral and tufted cells share many morphological properties. For example, they both extend a single primary dendrite to one of the several thousand glomeruli in the olfactory bulb. This observation signifies that each projection neuron receives odor information originating from only one type of odorant receptor. Therefore, even at the level of projection neurons, each neuron follows the “single cell – single odorant receptor” rule. Furthermore, neurons associated with the same glomerulus (sister cells) receive homogeneous input from the same olfactory sensory neurons and thus are thought to have similar odorant response properties. However, their odor-tuning specificity is variable, depending on odor concentration and spatial location (Tan et al., 2010; Kikuta et al., 2013). In addition, sister cells reportedly have various temporal activity patterns (Dhawale et al., 2010).

Mitral and tufted cells also share certain biophysical properties. For instance, the dendrodendritic reciprocal synapses between tufted and granule cells are both AMPA and NMDA receptor-mediated synapses but NMDA receptor is essential for the dendrodendritic inhibition (Christie et al., 2001), as are the synapses between mitral and granule cells (Isaacson and Strowbridge, 1998; Chen et al., 2000). Therefore, tufted cells tend to be regarded as smaller mitral cells, and they are often, especially in physiological studies, categorized into a single group termed “mitral/tufted cells.” In other organisms frequently used in olfactory research, such as the fly and the fish, mitral, and tufted cells are not morphologically well segregated and are accordingly categorized into a single group of projection neurons (Satou, 1990; Bargmann, 2006).

However, in the mammal, the morphologies of mitral and tufted cells are discriminable, especially by the location of the cell body and the extension pattern of secondary dendrites in the EPL (Mori et al., 1983; Orona et al., 1983). The secondary dendrites of the majority of tufted cells extend to the superficial/outer EPL, while those of mitral cells mostly extend to the deep/inner EPL. Hence, the EPL could be subdivided into at least two sublayers, the superficial/outer and the deep/inner layers, although a clear marker for the sublayers is not known. Interestingly, an early report identified a third intermediate sublayer, which is labeled by cytochrome oxidase (Mouradian and Scott, 1988).

Cell bodies of tufted cells are sparsely distributed in the EPL, and it is rare for them to find adjacent tufted cells. To the contrary, the cell bodies of mitral cells are surrounded by adjacent mitral cells in the MCL. Given the idea that more closely situated neurons tend to interact more strongly via reciprocal synapses with granule cells, such interactions are expected to be stronger among mitral cells than among tufted cells. The difference may, at least partially, be responsible for functional discrepancies between mitral and tufted cells (e.g., differential odor selectivity; Kikuta et al., 2013).

Targets of axons also differ between tufted cells and mitral cells (Haberly and Price, 1977; Skeen and Hall, 1977; Scott et al., 1980). The axons of tufted cells project to the anterior olfactory cortex, including the olfactory peduncle, olfactory tubercle, and ventrorostral subdivision of the piriform cortex. By contrast, those of mitral cells project widely throughout the entire olfactory cortex (Nagayama et al., 2010; Igarashi et al., 2012). Thus, there may be two types of axon bundles in the lateral olfactory tract, where one bundle is composed of thicker axons, and the other is composed of thinner axons [Price and Sprich, 1975; see also Bartolomei and Greer (1998) for PCD mice]. It is speculated that the thicker axons represent projections from mitral cells, while the thinner axons represent projections from tufted cells.

Functionally, tufted cells have lower thresholds to induce spike discharges by electrical stimulation of olfactory sensory neurons (Ezeh et al., 1993). The same result was also obtained with odorant stimulation of sensory neurons (Igarashi et al., 2012; Kikuta et al., 2013). In addition, tufted cells show a higher firing frequency than mitral cells (Nagayama et al., 2004), and tufted cells respond to a broader range of odorants than mitral cells (Nagayama et al., 2004; Kikuta et al., 2013). Recent reports also indicated that tufted cells respond during an earlier phase of the respiratory cycle, while mitral cells are activated during a later phase of the respiratory cycle (Fukunaga et al., 2012; Igarashi et al., 2012).

Mitral and tufted cells are generated during different periods of development. Whereas most mitral cells are born between embryonic days 10 and 13, tufted cells are born during a later period (embryonic days 13–16; Hinds, 1968; Imamura et al., 2011). The distinction in the timing of the genesis of tufted cells and mitral cells may affect to the differential locations of their somata, extension patterns of secondary dendrites, axon projections, and terminal locations (Inaki et al., 2004; Imamura et al., 2011).

Below, we discuss the structural and functional features of subgroups of tufted cells, mitral cells, and interneurons. The morphological features of these various kinds of neurons are summarized in **Figure 2B** and **Table 2**.

**Table 2:** External plexiform layer and mitral cell layer.

Projection neurons					
Cell type	Tufted cell		Mitral cell	
	Middle	Internal	Type-I	Type-II
Soma size	15–20 μm	>20 μm	>20 μm

Soma location	Superficial-intermediate EPL	Deep EPL	MCL

Dendrite extension	Superficial-intermediate EPL	Deep EPL	Intermediate EPL

Output within OB	Dendrites of PG, granule (probably type-I and -III) and other interneurons in EPL	Dendrites of PG, granule (Probably type-I and -II) and other interneurons in EPL

Output out of OB	Anterior part of olfactory cortex	Entire olfactory cortex

Transmitter	Glutamate

Molecular markers	Tbx21, Pcdh21

Population	~50 tufted cells/glomerulus	~20 mitral cells/glomerulus

Physiological properties	Higher sensitivity to the odor stimuli	Lower sensitivity to the odor stimuli

	Odor evoked spike activity during early phase of respiratory cycle	Odor evoked spike activity during late phase of respiratory cycle

Additional notes	Sometimes, it is called intermediate tufted cells	Sometimes, it is called displaced mitral cells		

References	Mori et al. (1983), Orona et al. (1984), Ezeh et al. (1993), Royet et al. (1998), Nakajima et al. (2001), Nagai et al. (2005), Shepherd et al. (2004), Igarashi et al. (2012), Fukunaga et al. (2012)			



**Interneurons**					
**Cell type**	**Van Gehuchten cell types**		**Multipolar types**		
	**Van Gehuchten cell**	**Somatostatin-immunoreactive cell**	**Inner horizontal cell**	**Other types**	**Large short axon cell**

Soma size	~12 μm	~10 μm	9–12 μm	9–15 μm	~14 μm

Soma location	Throughout the EPL	Intermediate-deep EPL	Deep EPL and MCL	Throughout the EPL	Superficial EPL

Dendrite extension	Throughout the EPL	Deep EPL	MCL and just above MCL	Throughout the EPL	EPL and GL

Output	*Unknown*	Probably mitral cell	probably mitral cell	Mitral/tufted cell	*Unknown*

Transmitter	Almost all of EPL interneurons are GABAergic				

Molecular markers	CR	Somatostatin, CR, VIP	CR

Axon	No				Yes

Additional notes	Fusiform shape soma				Relatively rare to be found

References	Schneider and Macrides (1978), Kosaka et al. (1994), Brinon et al. (1998), Kosaka and Kosaka (2008a), Lepousez et al. (2010)				

### SUBGROUPS OF TUFTED CELLS

Currently, tufted cells tend to be categorized into three subgroups: ET cells, middle tufted cells, and internal tufted cells. ET cells have relatively small cell bodies (10–15 μm) and are located around the boundary between the GL and the EPL (Pinching and Powell, 1971a). As discussed previously, ET cells can be separated into two distinct populations, one with no secondary dendrites, and the other with extended secondary dendrites in the superficial EPL.

Middle tufted cells are located in the intermediate and superficial EPL, which lies underneath the boundary between the GL and EPL. As might be expected, the cell body of these cells is of a medium size (15–20 μm; Shepherd et al., 2004). The majority of the middle tufted cells extend relatively short secondary dendrites in the superficial and intermediate EPL. They also extend axon collaterals in the IPL (Orona et al., 1984), which probably make contact with the other side (lateral-medial) of olfactory bulb like ET cells (Belluscio et al., 2002).

Internal tufted cells have relatively large cell bodies (>20 μm) and are located in the deep portion of the EPL (Orona et al., 1984). Internal tufted cells are sometimes called displaced mitral cells, because they reportedly have structural and functional properties similar to mitral cells. They also extend secondary dendrites in the intermediate and superficial EPL, as found with middle tufted cells. No tufted cells have been observed to extend secondary dendrites in the deep EPL.

### SUBGROUPS OF MITRAL CELLS

Mitral cells have large cell bodies (>20 μm) and are found in the MCL. The majority of these cells extend long secondary dendrites predominantly in the deep EPL and are termed type-I mitral cells (Mori et al., 1983; Orona et al., 1984). However, some mitral cells extend secondary dendrites predominantly in the intermediate EPL and are termed type-II mitral cells (Orona et al., 1984; Mouradian and Scott, 1988). In addition, subsets of mitral cells extend secondary dendrites in the superficial EPL (Imamura and Greer, unpublished data) and may not represent either type-I or type-II mitral cells. This new type of mitral cell may thus make a unique odor-processing contribution and receive dendritic inhibition in the superficial EPL (like tufted cells), as well as strong somatic inhibition in the MCL from type-S granule cells (also see GCL section). Recently, several reports have suggested the presence of different types of mitral cells based on their structural and functional properties (Padmanabhan and Urban, 2010; Angelo and Margrie, 2011; Angelo et al., 2012; Kikuta et al., 2013). By using *in vivo* two-photon imaging microscopy, mitral cells were recently grouped into three subtypes according to cell body shape: triangular, round, and fusiform type (Kikuta et al., 2013). Due to the lack of detailed evidence about the secondary dendrite extension pattern for each of these three subtypes, it is still unclear whether these cells are related to type-I or type-II mitral cells.

Mitral cells vary in molecular expression profiles. Subsets of the cells express the α3 subunit of the GABA_A_ receptor (Panzanelli et al., 2005), and variably express the voltage-gated potassium channel (e.g., Kv1.2) and the hyperpolarization-activated cyclic nucleotide gated channel (e.g., HCN2; Padmanabhan and Urban, 2010; Angelo and Margrie, 2011). Because HCN2 channel expression levels may be strongly associated with the parental glomerulus, olfactory sensory neuronal activity likely influences channel expression in mitral cells (Angelo et al., 2012). These data suggest the possibility that mitral cells can be subdivided based on the expression levels of specific molecules. Recent reports revealed that intrinsic biophysical properties also vary among mitral cells, such as firing frequency (Padmanabhan and Urban, 2010) and the *I*_h_ sag current (Angelo et al., 2012). The *I*_h_ sag current is probably associated with HCN2 expression levels. These studies highlight the possibility that the activity of mitral cells is controlled not only by inhibitory neurons in the olfactory bulb circuit, but also by intrinsic physiological properties.

As noted above, several reports indicate variations in mitral cell morphology, molecular expression profiles, and biophysical properties. However, it is uncertain whether these properties are related to one another. Connecting information and drawing a detailed profile of each mitral cell subtype will undoubtedly promote an understanding of odor coding.

### INTERNEURONS IN THE EXTERNAL PLEXIFORM LAYER AND MITRAL CELL LAYER

Several types of local neurons exist in the EPL (Schneider and Macrides, 1978; Shepherd et al., 2004). The majority are GABAergic neurons and make reciprocal synaptic contacts with mitral cells (Kosaka et al., 1994; Toida et al., 1994). These neurons reportedly express several calcium-binding proteins, such as PV, CB, CR, and neurocalcin (Brinon et al., 1999). Almost all EPL interneurons express CR. One-third are PV-positive neurons, which are well-studied, especially regarding their structural features (Lepousez et al., 2010; Huang et al., 2013). Nonetheless, PV is expressed in multiple morphological subtypes and thus, cannot be used as a definitive marker for a specific neuronal subtype. We first discuss PV-positive neurons below, and then briefly introduce several morphologically identified subtypes of EPL interneurons.

#### Parvalbumin-positive neurons

PV-positive cells in the EPL have quite varied structural properties. They can be categorized into five groups: sSA cells, Van Gehuchten cells, multipolar-type cells, inner short-axon cells, and inner horizontal cells (Kosaka et al., 1994). The latter two are regarded as particular subtypes or variations of the multipolar-type cell. Although some of the neurons are termed short-axon cells, it is not clear whether these PV-positive cells have axons or not (also see the following section, “short-axon cells in the external plexiform layer”; Kosaka et al., 1994; Kosaka and Kosaka, 2008a). PV-positive cells show reciprocal interactions with mitral cells, receiving excitatory inputs from mitral/tufted cells and returning inhibition to mitral/tufted cells, as seen with granule cells (Toida et al., 1994; Huang et al., 2013; Kato et al., 2013; Miyamichi et al., 2013). An interesting finding is that PV-positive cells have quite wide odorant selectivity, and odors can activate them in a broad area. Therefore, PV-positive neurons are expected to contribute to the non-specific gain control signals in the olfactory bulb circuit (Kato et al., 2013; Miyamichi et al., 2013).

A recent study reported that ∼80% of corticotropin-releasing hormone (CRH)-positive neurons express PV, and that the number of CRH-expressing cells is almost the same as the number of PV-positive cells in the EPL (Huang et al., 2013). This implies that the majority of CRH- and PV-expressing cells represent the same population. CRH-positive cells have a relatively low input resistance, a high capacitance, and a high firing rate response to current injection. Therefore, CRH-positive interneurons are considered to comprise a population of medium-sized and fast-spiking interneurons in the EPL (Huang et al., 2013).

#### Short-axon cells in the external plexiform layer

Historically, short-axon cells in the EPL were visualized by using Golgi techniques (Schneider and Macrides, 1978), and their structural details were studied by using PV immunostaining (Kosaka et al., 1994; Brinon et al., 1998). In some PV-positive cells, the axon initial segments of axon-like processes lack βIV-spectrin, an essential protein for spike propagation in axon. This observation suggests that the processes might not function as typical axons. Therefore, only large, multipolar cells with distinctive axons are defined as short-axon cells among PV-positive cells (Kosaka and Kosaka, 2008a). Interestingly, trans-synaptic tracing methods indicated that some of the short-axon cells in the EPL are presynaptic to adult-born neurons (Arenkiel et al., 2011). This implies that short-axon cell-like interneurons in this region may act to recruit or navigate newly generated granule cells into proper connections with mitral/tufted cells.

#### Van Gehuchten cells

This neuron type is considered an axonless cell. Van Gehuchten cells have medium-sized somata (∼12 μm) and few dendrites with polarized extension patterns. The thicker and longer dendrites (<150 μm) extend toward one side of the EPL, and the thinner and relatively shorter dendrites (<100 μm) extend toward the opposite pole (Schneider and Macrides, 1978). Half of these cells are PV-positive (Kosaka et al., 1994; Toida et al., 1996). Neurocalcin- or CB-positive cells are rarely found (Brinon et al., 1998).

#### Somatostatin-immunoreactive neurons

These neurons are recently identified and share some of the same morphological features as Van Gehuchten cells. They are also considered axonless cells. The soma size is approximately 10 μm. The majority of somatostatin-immunoreactive (SRIF-ir) neurons (∼95%) are located in the intermediate and deep EPL and extend dendrites specifically into the deep EPL (Lepousez et al., 2010). Because most mitral cells (type-I) extend secondary dendrites in the deep EPL, the SRIF-ir neurons may connect specifically with mitral cell secondary dendrites. SRIF-ir neurons are also GABAergic (99.4%), CR-positive (99.9%), and vasoactive intestinal polypeptide (VIP)-positive (96.7%) cells. Half of these cells are also PV-positive.

## GRANULE CELL LAYER

The GCL of the olfactory bulb is occupied mostly by granule cells, which are inhibitory interneurons with a small cell body (6–8 μm in diameter; Price and Powell, 1970b). Their somata are localized mostly in the GCL, but some granule cells are also found in the IPL and the MCL. An apical dendrite typically extends radially toward the surface of the olfactory bulb and rarely branches in the GCL, until it ramifies in the EPL. In the EPL, granule cells have dendritic spines that form reciprocal synapses with the secondary dendrites of mitral and/or tufted cells. Due to their axonless morphology, the output of granule cells relies solely on dendrodendritic synapses.

The other interneurons in the GCL are deep short-axon (dSA) cells. Short-axon cells comprise heterogeneous populations, and they were previously classified into multiple subpopulations based on their soma location and morphology (Schneider and Macrides, 1978; Shepherd et al., 2004). Among these short-axon cells, the Blanes cells, Golgi cells, Cajal cells, and horizontal cells have their cell bodies in the deeper regions of the olfactory bulb, including the MCL, IPL, and GCL. These cells are now collectively re-classified as dSA cells (Eyre et al., 2008). Compared with granule cells, dSA cells have larger cell bodies (10–20 μm in diameter), and their dendrites do not usually extend beyond the MCL. On the other hand, they have axons that project to different layers of the olfactory bulb (see below).

Granule cells and dSA cells are divided into subgroups, mostly based on morphological differences between the groups. These cells are discussed in detail below, and their properties are summarized in **Figure 2C** and **Table 3**.

**Table 3:** Granule cell layer.

Granule cell						
Cell type	Type-I	Type-II	Type-III	Type-IV (deep-branching)	Type-V (shrub)	Type-S
Soma size	6–8 μm					

Soma location (primary)	*Unspecified*	Deep GCL	Superficial GCL to MCL	*Unspecified*	MCL	Middle GCL

Dendrite extension	All through EPL	Deep EPL	Superficial EPL	Frequently branch in GCL	Deep EPL (no basal dendrite)	MCL

Transmitter	GABA			GABA?		GABA

Output	Mitral/tufted cell	Mitral cell?	Tufted cell?	*Unknown*	Mitral cell?	Mitral cell soma

References	Mori et al. (1983), Orona et al. (1983)			Merkle et al. (2014)		Naritsuka et al. (2009)

Deep short-axon cell						
**Cell type**	**GL-dSA**		**EPL-dSA**		**GCL-dSA**	

Soma size	14–20 μm		11–15 μm		10–20 μm	

Soma location (primary)	IPL		GCL		GCL	

Dendrite extension	Predominantly confined to the IPL		MCL, IPL, GCL		MCL, IPL, GCL	
Axon extension	GL (some in EPL)		EPL (some in IPL and superficial GCL)		GCL (some to the olfactory cortex)	

Output	PG cell (granule cell)		Granule cell		Granule cell	

Transmitter	GABA				

Additional notes	Horizontal cells, Golgi cells		Blanes cells, Cajal cells		Horizontal cells, Golgi cells	

References	Schneider and Macrides (1978), Eyre et al. (2008, 2009)					

### MORPHOLOGICAL DIVERSITY OF GRANULE CELLS

Intracellular horseradish peroxidase (HRP) injection into the rabbit olfactory bulb cells revealed at least three morphologically distinct subpopulations of granule cells based on their dendritic extension patterns in the EPL (Mori et al., 1983; Mori, 1987). The type-I granule cell (GI) ramifies spiny dendrites at any depth of the EPL. Dendrites of the type-II granule cell (GII) extend only in the deep EPL, while the type-III granule cell (GIII) ramifies spiny dendrites predominantly in the superficial EPL. Therefore, type-II and type-III granule cells are thought to preferentially regulate the activity of mitral cells and tufted cells, respectively. In an early work, Orona et al. (1983) injected HRP into the EPL of the rat olfactory bulb to label granule cells extending dendrites to the injection site. Injection of HRP into the superficial EPL preferentially labeled the granule cells localized in the superficial portion of the GCL (superficial granule cells). In contrast, HRP injection into the deep EPL labeled additional granule cells in the deep GCL (deep granule cells). These results strongly indicate a correlation between somata location and dendrite-ramifying area. Nonetheless, the location of somata is not a definitive determinant of type-I, II, or III cells, because some superficial granule cells ramified dendrites in the deep EPL, while some deep granule cells sent dendrites up to the superficial EPL.

Several other types of granule cells do not fit into type-I, II, or III categories. Naritsuka et al. (2009) discovered a novel type of cell in the GCL of a transgenic mouse expressing GFP under the control of the nestin promoter. The cell was named a type-S cell based on its strong GFP expression. Although neuronal progenitor cells usually express nestin, the type-S cell was defined as a mature neuron due to its expression of NeuN, a marker of mature neurons, and the existence of dendritic spines. The cell was also considered a granule cell based on its GABAergic phenotype and axonless morphology. The especially unique feature of the type-S granule cell was that the apical dendrites did not penetrate into the EPL, but rather formed reciprocal synapses with the perisomatic region of mitral cells. Therefore, the dendrites might regulate the production of action potentials in mitral cells. This hypothesis could be tested by experiments using electrophysiology and/or optogenetics.

Recently, Merkle et al. (2014) reported four previously unknown interneuron types that are generated in the adult mouse ventricular zone-SVZ and migrate to the GCL. Based on soma size/location, dendritic arbors bearing spines, and axonless morphology, two of the cells were termed type-IV granule cells (GIV) and type-V granule cells (GV). The type-IV granule cell was frequent dendritic branching in the GCL, and often failed to reach beyond the IPL. Somata of type-V granule cells lacked basal dendrites and were restricted to the MCL. These cells extended spiny shrub-like apical dendrites predominantly into the deep EPL. Due to their characteristic features, the authors also called type-IV and -V granule cells deep-branching granule cells and shrub granule cells, respectively. However, neurotransmitter and existence of reciprocal synapses are still necessary to be specified, which will increase our knowledge of granule cell diversity.

### MOLECULAR DIVERSITY OF GRANULE CELLS

To date, only GABA has been identified as a neurotransmitter of the granule cell. Although glycine evokes an inhibitory response in both mitral/tufted cells and granule cells, and immunoreactivity for glycine, the glycine receptor, and glycine transporters is found in the olfactory bulb (van Den Pol and Gorcs, 1988; Trombley and Shepherd, 1994), there is no direct evidence suggesting the existence of glycinergic neurons in the GCL (Zeilhofer et al., 2005). Therefore, glycinergic axons may originate outside the olfactory bulb.

In contrast to PG cells, granule cells are molecularly less diverse. Among known molecular markers expressed by subpopulations of PG cells (i.e., TH, CR, CB, and PV), only CR is expressed by a subset of granule cells that are localized in superficial GCL (Batista-Brito et al., 2008). In addition, 5T4, a leucine-rich-repeat transmembrane protein, also labels a subpopulation of superficial granule cells (Imamura et al., 2006; Yoshihara et al., 2012). Because dendrites of the 5T4-positive granule cells preferentially ramify in the superficial EPL, these cells most likely represent type-III granule cells. On the other hand, 5T4-positive granule cells are mostly found in the MCL and IPL, but rarely in the GCL, suggesting that they account for only a subset of type-III granule cells.

Curiously, few molecules specifically expressed by deep granule cells have been identified. As an exception, electrophysiological analyses suggested that expression levels of Group I metabotropic glutamate receptors (mGluRs) differed between superficial and deep granule cells (Heinbockel et al., 2007). (RS)-3,5-dihydroxyphenylglycine (DHPG), an agonist of Group I mGluRs, directly depolarized both superficial and deep granule cells in the wild-type mouse olfactory bulb slice. In mGluR5^-/-^ and mGluR1^-/-^ mice, only superficial and deep granule cells, respectively, were depolarized with DHPG. Further confirmation of differential expression of these molecules could be achieved by immunohistochemical analyses with antibodies specific to mGluR1 and mGluR5.

Recently, Saino-Saito et al. (2007) reported that CaM kinase IV (CaMKIV) expression is restricted to deep granule cells. However, according to the immunohistochemical staining pattern, CaMKIV-positive cells localized to the entire GCL, but rarely to the MCL. The authors apparently defined granule cells in the whole GCL as deep granule cells. Therefore, albeit an interesting observation, CaMKIV might not be specific to deep granule cells under the definition in this review, in which the GCL is divided into superficial and deep layers. One possible reason for the lack of identification of any molecule specifically expressed by deep granule cells is simply that we have not yet examined the expression of the appropriate molecules. However, an alternative possibility is that granule cells innately have properties to differentiate into deep granule cells, and are thus redirected toward superficial granule cells by the expression of alternative molecules, such as Pax6 (see below).

### ADULT-BORN GRANULE CELLS

An estimated 10,000 new neurons are generated in the adult mouse SVZ and enter into the olfactory bulb via the rostral migratory stream every day. Approximately half of these new neurons are integrated into the existing neuronal circuit as adult-born granule cells (Petreanu and Alvarez-Buylla, 2002; Yamaguchi and Mori, 2005). On the other hand, many pre-existing granule cells are eliminated from the circuit via apoptotic cell death every day. Whether specific types of granule cells are replaced with adult-born granule cells is an interesting question. Both morphologically and molecularly, all types of granule cells mentioned above, except for type-S cells, are generated in the adult olfactory bulb (Merkle et al., 2007, 2014). To date, the replacement of type-S cells has not been investigated. Although adult-born granule cells are distributed throughout the MCL, IPL, and GCL, several studies have suggested that adult-born granule cells are located in the deep GCL with higher density, while embryonically/perinatally born granule cells are preferentially located in the MCL, IPL, and the superficial GCL (Lemasson et al., 2005; Imayoshi et al., 2008). Intriguingly, retroviral fate mapping in the rat revealed that more adult-born granule cells ramified their dendrites in the deep EPL, even though their somata were located in the superficial GCL (Kelsch et al., 2007). These studies raise the idea that the microcircuit formed in the deep EPL is more plastic than that in the superficial EPL in the adult olfactory bulb.

### DETERMINANTS OF GRANULE CELL DIVERSITY

What determines the final morphology and molecular phenotype of granule cells? Deep granule cells are mainly generated during postnatal stages, while superficial granule cells have a generation peak around the perinatal period (Hinds, 1968). Thus, the timing of neurogenesis influences the fate of granule cells. However, the factor that probably has the largest influence on granule cell diversity is the SVZ region in which the granule cell is generated. Neural stem cells in different portions of the SVZ produce different types of granule cells. Lineage tracing analyses of cells generated in various SVZ regions revealed that superficial and deep granule cells are preferentially produced in the dorsal and ventral SVZ, respectively (Merkle et al., 2007). Kelsch et al. (2007) used retroviral injection into the SVZ to show that the anterior and posterior axis also influenced the production of superficial and deep granule cells. In addition, Merkle et al. (2007) showed that CR-positive granule cells were mostly produced from the rostral migratory stream or the medial wall of the anterior SVZ. Furthermore, grafting experiments from these groups suggested that SVZ stem cells are highly resistant to respecification by environmental cues.

The molecular mechanisms regulating the production of specific granule cell types are not well known. Two transcription factors, Pax6 and ER81, are reportedly expressed only in subsets of superficial granule cells (Kohwi et al., 2005; Saino-Saito et al., 2007; Haba et al., 2009). Moreover, Pax6-deficient stem cells grafted into the wild-type SVZ produced many deep granule cells, but failed to produce superficial granule cells or TH-positive PG cells (Kohwi et al., 2005). These results may indicate that newly generated granule cells have innate properties to differentiate into deep granule cells, and are directed to become superficial granule cells via the expression of an additional set of transcription factors.

### DIVERSITY OF DEEP SHORT-AXON CELLS

Based on morphological diversity revealed by Golgi staining, dSA cells in the rodent olfactory bulb were initially defined belonging to one of four cell types: Blanes cells, Golgi cells, horizontal cells, and Cajal cells. Schneider and Macrides (1978) described the morphology and location of the various dSA cells in the hamster olfactory bulb. Blanes cells are mostly found in the GCL or the IPL and have the largest cell body (16–23 μm) of the four dSA cells. They also have stellate dendrites covered with many spines. Golgi cells likewise are found in the GCL. Their cell bodies are slightly smaller than those of Blanes cells (12–22 μm), and their dendrites rarely have spines. Both horizontal cells and Cajal cells have the smallest cell bodies (15–18 μm), are restricted to the IPL and MCL, and have smooth dendrites. To date, all dSA cells are considered to be GABAergic, as are their post-synaptic target cells. Recently, dSA cell morphologies were reconstructed after electrophysiological recording in a rat olfactory bulb slice (Eyre et al., 2008). Overall, the dendrites of all four dSA cell types were restricted to the layers below the MCL. Despite the Schneider and Macrides report of the extension of Cajal cell dendrites to the EPL, this was not described by Eyre et al. Although the input sources were not revealed in detail, both excitatory and inhibitory inputs apparently modulated the activity of dSA cells.

Eyre et al. (2008) next re-classified dSA cells into three subpopulations according to their axonal distribution patterns in the olfactory bulb. The first subpopulation corresponds to the GL-dSA cell. The axons of these cells travel in the EPL up to the GL, where they extensively ramify with a few axon collaterals extending into the EPL and GCL. The major post-synaptic targets of GL-dSA cells are PG cells in the GL, while symmetrical synapses between axons of GL-dSA cells and the dendrites of granule cells are found in the EPL and GCL. The somata of many GL-dSA cells elongate in a direction parallel to the MCL, and are mostly found in the MCL and IPL. The dendrites of these cells are predominantly confined to the IPL. According to the authors, GL-dSA cells have an overall appearance of horizontal cells or Golgi cells.

The second re-classified dSA cell population is the EPL-dSA cell. Axons of EPL-dSA cells ramify predominantly within the EPL, and never enter the GL. Their somata are mostly found in the GCL, and the majority have stellate or vertically oriented dendrites with spines. However, some EPL-dSA cell dendrites are devoid of spines. Morphological features suggest that the bulk of EPL-dSA cells are Blanes cells, along with some Cajal cells. The post-synaptic targets of EPL-dSA cells in the EPL are the dendritic shaft and spines of granule cells, but some synapses are also found in the GCL. Pressler and Strowbridge (2006) showed that Blanes cells monosynaptically inhibited granule cells, and that the persisting firing of Blanes cell caused long-lasting inhibition of granule cells.

The third re-classified population is the GCL-dSA cell. Axonal arbors of GCL-dSA cells are mostly restricted to the GCL and contact the proximal dendrites and somata of granule cells. Some axons also project to the olfactory cortices. The dendrites of these cells are usually sparsely spiny. Eyre et al. (2008) described GCL-dSA cells as having an overall appearance of horizontal cells or Golgi cells.

Certain molecules, such as neuropeptide Y (Gall et al., 1986), CB (Brinon et al., 1992), VIP (Gracia-Llanes et al., 2003), neuronal nitric oxide synthase (nNOS) (Kosaka and Kosaka, 2007b), and neurocalcin (Brinon et al., 1998), are expressed by subpopulations of dSA cells. However, dSA cell subtypes that express these molecules have not been well identified. Assorted dSA cells seem to have divergent electrophysiological properties, which is probably related to the differential expression of channels and neurotransmitter receptors (Eyre et al., 2008, 2009).

## SUMMARY

### THE OLFACTORY BULB CIRCUIT AS BASED ON RECENTLY IDENTIFIED NEURONAL GROUPS

**FIGURE 3:**
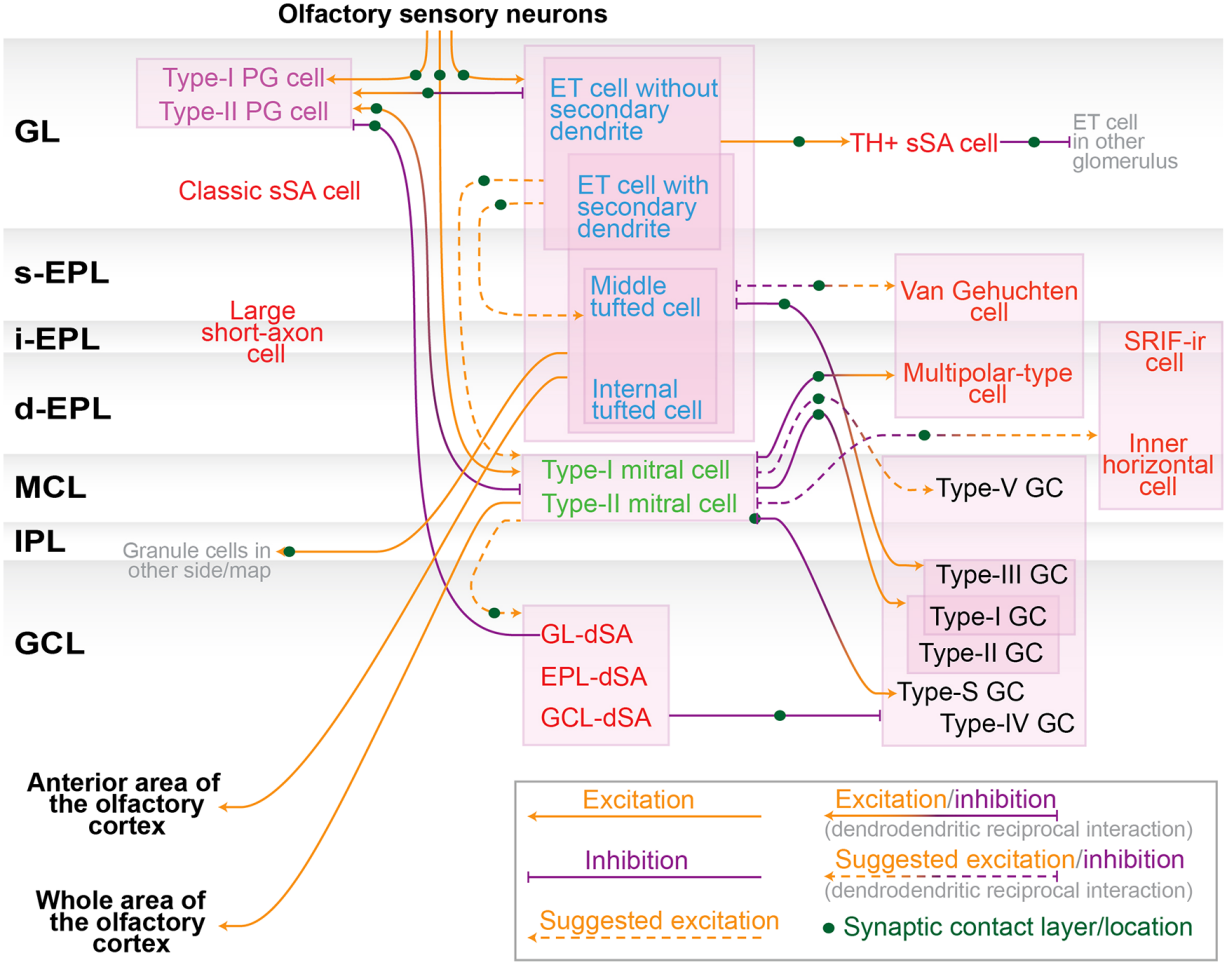
**Neuronal connection in the olfactory bulb.** Schematic diagram of the neuronal synaptic connection among nerve terminal of olfactory sensory neurons, subtypes of periglomerular (PG) cells (purple), tufted cells (blue), mitral cells (green), granule (GC) cells (black), and other interneurons including various short-axon cells (red). These colors are consistent with previous figures. Orange and purple arrows represent excitatory and inhibitory inputs, respectively. Dendrodendritic excitatory/inhibitory interaction is represented by orange/purple bidirectional arrows. Connections suggested from the dendritic arborization, axonal projection pattern and physiological data are shown in dashed lines. Several neuron types are left unwired due to lack of sufficient information. Note that there are interactions not appeared in this diagram, including electric couplings between divergent neurons (e.g., mitral–mitral, mitral–tufted, tufted–tufted and mitral–interneurons; Kosaka and Kosaka, 2003; Christie et al., 2005; Hayar et al., 2005; Kosaka et al., 2005; Ma and Lowe, 2010), spillover of glutamate (Aroniadou-Anderjaska et al., 1999; Carlson et al., 2000), GABA (Aroniadou-Anderjaska et al., 2000; Smith and Jahr, 2002; Murphy et al., 2005) and dopamine (Ennis et al., 2001). Abbreviations: dSA, deep short-axon; GL, glomerular layer; s/i/d-EPL, superficial/intermediate/deep external plexiform layer; MCL, mitral cell layer; IPL, internal plexiform layer.

This review summarizes the properties of numerous neurons in the olfactory bulb by focusing on their morphology, protein expression, and function. As the motivation behind this work is to have a more advanced model of olfactory bulb circuit, we assembled the recognized neuronal connections into a diagram (**Figure 3**). We included the connections suggested from the dendritic arborization, axonal projection pattern and physiological data in addition to the established connections. Although the diagram is still far from complete, it would be helpful for neuronal researches in the olfactory bulb circuits. In addition, we discuss some of the issues not highlighted above to bridge the recent research results and the conventional diagram of the circuit.

#### External tufted cells and superficial tufted cells

In the course of defining the neuronal groups, the categorization of ET cells and superficial tufted cells creates some confusion. More specifically, there seems to be little consensus regarding the exact definition of a superficial tufted cell. In literature, the term superficial tufted cell could be just a synonym of ET cell, or it could represent a tufted cell whose cell body is located in the superficial part of the EPL (Kosaka et al., 1998; Luo and Katz, 2001; Hamilton et al., 2005). As discussed in the GL section, ET cells consist of two subtypes that are morphologically and physiologically distinct. In some reports, the superficial tufted cell is described as the subtype with secondary dendrites and therefore the term ET cell only represents the subtype without secondary dendrites (Ezeh et al., 1993; Kiyokage et al., 2010). In our opinion, this seems the most sensible use of the terms.

A fraction of putative middle tufted cells located in the superficial EPL potentially belongs to the superficial tufted cells as well. Both of two ET cell/superficial tufted cell subpopulations that release peptide hormones CCK and vasopressin, respectively (Liu and Shipley, 1994; Tobin et al., 2010), have secondary dendrites and are thus most likely involved in the superficial tufted cells, implying the further functional heterogeneity of superficial tufted cells. Additional investigations are required to definitively conclude how superficial tufted cells differ from ET cells (i.e., without secondary dendrites) or from middle tufted cells. For example, it is an interesting question whether the targets of their axon and/or axon collaterals are the same, partially overlapping, or mutually exclusive.

#### Tufted cells and mitral cells

Here, we regard mitral and tufted cells as different cell types due to their divergent properties, including the location of the cell body, patterns of secondary dendrite extension and axonal projection, firing frequency, odor sensitivity, tuning specificity, and odor response phase on the respiratory rhythm. However, as stated above, recent investigations have identified certain projection neurons that have features of both tufted and mitral cells, such as mitral cells that extend secondary dendrites into the superficial EPL. These reports raise the question of whether mitral and tufted cells actually compose segregated circuits, and whether they are clearly distinguishable cell types or not.

A provocative idea is that mitral and tufted cells are on a continuum of neurons physically aligned from the superficial to deep portions of the EPL–MCL area, where the continuum comprises ET, superficial tufted, middle tufted, internal tufted, type-II mitral, and type-I mitral cells. The alignment may be also associated with the birth date of the neurons during the embryonic stage. A key point to examine within this hypothesis is the existence of any molecule(s) with a differential expression pattern between mitral and tufted cells.

#### Granule cells

This review describes morphologically distinct granule cells in the rodent olfactory bulb, particularly in regard to dendritic extension patterns in the EPL. In fact, at least three subgroups of granule cells have been identified according to dendritic morphology: type-I, II, and III granule cells. However, these three subtypes do not cover the entire granule cell population, because some granule cells have morphologies that do not resemble any of these types. One example is the type-S cell. More recently, Merkle et al. (2014) reported novel granule cells in the GCL: the type-IV and V granule cells. There are possibly more unidentified granule cells in the GCL. Again, the identification of any molecules that could segregate the different types of granule cells is a question of interest for future exploration.

Another unanswered question is whether different types of granule cells have divergent functions in the neuronal network in the olfactory bulb. Very limited information is available about the electrophysiological properties, target cells, and responses to odorants among various granule cells. For example, the dendritic morphologies in the EPL suggest that type-II and III granule cells regulate mostly mitral and tufted cells, respectively, although this hypothesis has not yet been tested.

Also unknown is the matter of any functional structure in the GCL. Recent explorations have used virus tracing methods to show that the GCL has a columnar structure (Willhite et al., 2006). Although it is not known whether the columnar structure of the GCL reflects the spatial pattern of a group of neurons strongly connected to each other or the expression of specific synaptic molecules that are essential for trans-synaptic virus infection, this observation lends support to the idea that the GCL may indeed have a functional structure. Future research to uncover the organization of the GCL will be an important challenge to more clearly understand the olfactory bulb circuit.

#### Short-axon cells

One might consider that interneurons in the olfactory bulb other than PG cells and granule cells are the same type of neuron, since they are all called short-axon cells. Nonetheless, short-axon cells are a diverse population with varied morphologies and functions, and, thus, they should not be regarded as a single group. In addition, as discussed in the EPL section, some EPL interneurons, which were conventionally categorized as short-axon cells, may not have an actual axon, but instead have only an axon-like process (Kosaka and Kosaka, 2008a). These various short-axon cells deserve re-investigation with the aid of contemporary technology, similar to the recent re-investigations of dSA cells (Eyre et al., 2008, 2009).

#### Horizontal interactions in the olfactory bulb

The remarkable change from the conventional to updated olfactory bulb circuit diagram is that various types of cells can contribute to horizontal interactions in the GL and EPL. Conventionally, horizontal interactions were thought to be mediated mainly by dendrodendritic reciprocal synapses formed between mitral/tufted cells and granule cells. However, recent work revealed that sSA cells in the GL and PV-positive neurons in the EPL also contribute to horizontal interactions in each layer. Interestingly, the locations of the interaction sites are clearly segregated. The sSA cells mediate horizontal interactions only in the GL, while PV-positive cells mediate such interactions only in the EPL. Moreover, these cells differ in morphology. While the sSA cell has an axon, most PV-positive cells are axonless and send outputs from dendrites alone, similar to granule cells (Huang et al., 2013; Kato et al., 2013; Miyamichi et al., 2013). The locational and morphological variances probably reflect the functional distinctions between these neurons. Further clarification of such functional distinctions is essential for understanding how convergent odor information is processed through the interaction of multiple glomerular modules.

### CONCLUDING REMARKS

In general, recent studies in neuroscience have increasingly revealed the intricacy and complexity of the olfactory system, providing more a detailed, albeit a more complicated, diagram of the olfactory bulb network. However, many issues must still be addressed to accurately categorize neuronal types and to complete a comprehensive diagram of this network. The rapid progress in developing new techniques and technologies (e.g., *in vivo* two-photon imaging, CLARITY) is essential and quite helpful in overcoming some of the challenges that we still face in understanding the structure and function of neuronal networks with single cell resolution. Steady progress in characterizing each neuronal type along the full spectrum of its properties is one of our most immediate needs. Ultimately, as we dissect and begin to understand the detailed nature of the olfactory circuit networks, our next questions must focus on understanding how odorants within these circuits play a role in regulating behavior.

## Conflict of Interest Statement

The authors declare that the research was conducted in the absence of any commercial or financial relationships that could be construed as a potential conflict of interest.

## ABBREVIATIONS

*Brain areas*: AON: anterior olfactory nucleus
AONpE: anterior olfactory nucleus pars externa
SVZ: subventicular zone
*Layers*: ONL: olfactory nerve layer
GL: glomerular layer
EPL: external plexiform layer
s-EPL: superficial EPL
i-EPL: intermediate EPL
d-EPL: deep EPL
MCL: mitral cell layer
IPL: internal plexiform layer
GCL: granule cell layer
*Cells*: JG cell: juxtaglomerular cell
PG cell: periglomerular cell
ET cell: external tufted cell
sSA cell: superficial short-axon cell
dSA cell: deep short-axon cell
SRIF-ir cell: somatostatinimmunoreactive cell
*Molecules*: BrdU: 5-bromo-2′-deoxyuridine
CaMKIV: CaM kinase IV
CB: calbindin
CCK: cholecystokinin
CR: calretinin
CRH: corticotropin-releasing hormone
DHPG: (RS)-3,5-dihydroxyphenylglycine
GAD: glutamic acid decarboxylase
GFP: green fluorescent protein
HCN: hyperpolarization-activated cyclic nucleotide gated channel
HRP: horseradish peroxidase
Kv: voltage-gated potassium channel
mGluRs: metabotropic glutamate receptors
nNOS: neuronal nitric oxide synthase
PV: parvalbumin
TH: tyrosine hydroxylase
VGAT: vesicular GABA transporter
VGLUT: vesicular glutamate transporter
VIP: vasoactive intestinal polypeptide.
